# Artificial Intelligence vs. Human Cognition: A Comparative Analysis of ChatGPT and Candidates Sitting the European Board of Ophthalmology Diploma Examination

**DOI:** 10.3390/vision9020031

**Published:** 2025-04-09

**Authors:** Anna P. Maino, Jakub Klikowski, Brendan Strong, Wahid Ghaffari, Michał Woźniak, Tristan Bourcier, Andrzej Grzybowski

**Affiliations:** 1 Manchester Royal Eye Hospital, Manchester M13 9WL, UK; anna.maino@nhs.net; 2 Department of Systems and Computer Networks, Wrocław University of Science and Technology, 50-370 Wrocław, Poland; jakub.klikowski@pwr.edu.pl (J.K.); michal.wozniak@pwr.edu.pl (M.W.); 3 European Board of Ophthalmology Examination Headquarters, RP56PT10 Kilcullen, Ireland; brendan.strong@fs-examservices.ie; 4 Department of Medical Education, Stockport NHS Foundation Trust, Stockport SK2 7JE, UK; w.ghaffari@doctors.org.uk; 5 Department of Ophthalmology, University of Strasbourg, 67081 Strasbourg, France; tristan.bourcier@chru-strasbourg.fr; 6 Institute for Research in Ophthalmology, 60-836 Poznań, Poland

**Keywords:** artificial intelligence, ophthalmology, medical examinations

## Abstract

Background/Objectives: This paper aims to assess ChatGPT’s performance in answering European Board of Ophthalmology Diploma (EBOD) examination papers and to compare these results to pass benchmarks and candidate results. Methods: This cross-sectional study used a sample of past exam papers from 2012, 2013, 2020–2023 EBOD examinations. This study analyzed ChatGPT’s responses to 440 multiple choice questions (MCQs), each containing five true/false statements (2200 statements in total) and 48 single best answer (SBA) questions. Results: ChatGPT, for MCQs, scored on average 64.39%. ChatGPT’s strongest metric performance for MCQs was precision (68.76%). ChatGPT performed best at answering pathology MCQs (Grubbs test *p* < 0.05). Optics and refraction had the lowest-scoring MCQ performance across all metrics. ChatGPT-3.5 Turbo performed worse than human candidates and ChatGPT-4o on easy questions (75% vs. 100% accuracy) but outperformed humans and ChatGPT-4o on challenging questions (50% vs. 28% accuracy). ChatGPT’s SBA performance averaged 28.43%, with the highest score and strongest performance in precision (29.36%). Pathology SBA questions were consistently the lowest-scoring topic across most metrics. ChatGPT demonstrated a nonsignificant tendency to select option 1 more frequently (*p* = 0.19). When answering SBAs, human candidates scored higher than ChatGPT in all metric areas measured. Conclusions: ChatGPT performed stronger for true/false questions, scoring a pass mark in most instances. Performance was poorer for SBA questions, suggesting that ChatGPT’s ability in information retrieval is better than that in knowledge integration. ChatGPT could become a valuable tool in ophthalmic education, allowing exam boards to test their exam papers to ensure they are pitched at the right level, marking open-ended questions and providing detailed feedback.

## 1. Introduction

Neural networks were first described by McCulloch and Pitts in 1943, when they proposed the first mathematical model of a neuron [1]. They used an idea presented in Alan Turing’s work [2] and depicted how the brain works as a highly efficient computational system consisting of interconnected simple elements (so-called McCulloch–Pitts neurons) [1]. In 1986, Rumelhart et al. proposed a back-propagation algorithm that allowed multilayer neural networks to be trained efficiently and allowed these models to be used to solve real, nontrivial decision tasks [3]. However, only when massive computation was developed did deep neural networks (DNNs) become possible, bringing us closer to achieving a so-called human-level AI, also known as artificial general intelligence [3]. One of the fastest-growing trends is large language models (LLMs), which are DNNs based on so-called transformers and use huge language corpora for learning. These models are dedicated to natural language processing (NLP), making recognizing, translating, predicting, or generating text possible [4]. The most well-known LLM is ChatGPT (Chat Generative Pretrained Transformer, OpenAI, San Francisco, USA), a large-language-model-based chatbot that can process data into human-like text-based outputs. We selected ChatGPT as it was the most widely accessible free-to-use chatbot that could conduct web searches in 2023. Medical education is just one of the many fields that AI can revolutionize, from encouraging personalized learning to delivering adaptive educational content that addresses individual knowledge gaps to giving learners the possibility to study outside traditional settings [5]. On the other hand, there are also limitations that need addressing if AI is to be fully embraced by medical education, including transparency of the AI decision-making process, discrimination and bias due to insufficient training data [6], and the urgent need for learners, educators, and exam boards to be trained in how to utilize AI in teaching and evaluation [7].

Several studies have explored the use of AI in answering practice questions for medical examinations, and this is set to increase as AI becomes more widely used [8,9,10,11,12,13,14]. While ChatGPT cannot be considered a standalone, reliable source for ophthalmic education because of its limitations in data sources and knowledge cutoff, it offers accessibility, supplemental learning, interactive learning, and collaborative learning [15].

A main limitation of past studies is that they were conducted on extracted questions from question banks or from the web, while our aim was to conduct a study on original and complete sets of questions, comparing AI performance against that of hundreds of humans answering questions in real exam conditions.

The European Board of Ophthalmology (EBO) is a permanent working group of the ophthalmology subspecialty section of the European Union of Medical Specialists (UEMS). The EBO is tasked with harmonizing the standards of ophthalmology training across Europe. The EBO Diploma (EBOD) examination assesses the knowledge and clinical skills required to deliver high standards of ophthalmic care. The EBOD exam is aimed at eye doctors near the completion of their specialist training. It consists of a written part and a viva voce. Until 2021, the written part consisted of 52 text-based multiple-choice questions (MCQs). Each MCQ contained five statements that had to be independently marked as true or false, for a total of 260 statements per paper. The format changed in 2022, with fewer MCQs (44 questions, for a total of 220 statements) and the addition of 16 single best answers (SBAs). SBA questions offer four options, only one of which is the best answer. We did not administer viva voce questions to ChatGPT because these questions became standardized in 2021, making comparisons with previous years difficult.

This study aims to assess the performance of two ChatGPT models in answering previous EBOD examination questions and to compare these results with past benchmarks and candidate results. We also compared ChatGPT’s performance in answering different question formats (MCQs versus SBAs), and we investigated whether ChatGPT had identifiable biases or learning effects when answering questions. These aspects have to be investigated before adopting AI as a digital learning companion or utilizing it to mark examination papers.

## 2. Materials and Methods

This cross-sectional study used real past exam papers from the2012, 2013, 2020, 2021, 2022, and 2023 EBODs. We chose these years to avoid having the same question present in more than one paper. The authors obtained approval from the EBO Executive Committee before using the papers. The Department of Systems and Computer Networks, Wrocław University of Science and Technology, Poland, conducted the study between July and October 2023 using ChatGPT-3.5 Turbo and in January 2025 using ChatGPT-4o [16].

### 2.1. Prompt Engineering

Prompt engineering has a significant impact on generative LLM output [13]. We used a brand-new account without previous exposure to EBO exam questions. All MCQ questions were based on narrative text, so they were all included in the study (2200 questions).

For SBA questions, we excluded 4 questions containing images or tables, so we had a total of 44 SBAs. All SBA questions had the same format (clinical scenario followed by direct question, separated by a new line). The original set included 44 SBAs, so in order to increase the robustness of our data analysis and to be able to detect bias, we created a randomized set where were repeated the same SBAs 10 times, changing the order of the questions within the paper and the order of the 4 possible answers at random (randomized set, 440 questions).

### 2.2. Quality Metrics

The performance metrics were calculated based on the so-called confusion matrix, which summarizes the number of correctly and incorrectly classified instances in each class [17]. The most popular metrics measuring classifier performance are accuracy (how often ChatGPT is correct overall), recall (how complete ChatGPT’s correct prediction are), precision (how accurate ChatGPT’s correct answers are), and F1, an aggregate measure calculated using the first harmonic mean of recall and precision [18].

### 2.3. Secondary Outcomes

Secondary outcomes included exploring whether repeating test papers from different years affected the metric scores and whether ChatGPT was biased towards a particular question category. For the latter, we divided all exam questions according to 12 categories (A1: Optics and Refraction and Strabismus; A2: Pediatric Ophthalmology and Neuro-Ophthalmology; B1: Cornea; B2: Oculoplastics; C1: Glaucoma; C2: Cataract; D1: Retina; D2: Uveitis; General Medicine; Pathology; Pharmacology; Diagnostics; and Imaging).

We compared ChatGPT answers with those given by 2935 candidates. Moreover, from a random sample of 50 MCQ questions (250 statements), we selected the top 10 easiest and the top 10 most difficult questions based on the percentage of correct answers given by human candidates to investigate how our ChatGPT models performed when answering “easy” or “difficult” questions.

### 2.4. Statistical Analysis

Data were analyzed using MS Excel and GraphPad software (GraphPad QuickCalcs, Boston, MA, USA http://www.graphpad.com/quickcalcs/grubbs1.cfm, accessed on 4 January 2025). Continuous data were analyzed using linear regression and R-squared values to express the goodness of fit. We used the Grubbs test to identify outliers because we had a relatively small numbers of categories and only 1–2 outliers. Categorical variables were analyzed using contingency tables. *p*-values of less than 0.05 were considered statistically significant.

## 3. Results

This study analyzed ChatGPT responses to 392 MCQs (1432 statements) and 48 SBAs.

### 3.1. Multiple Choice Questions (MCQs)

**Figure 1:**
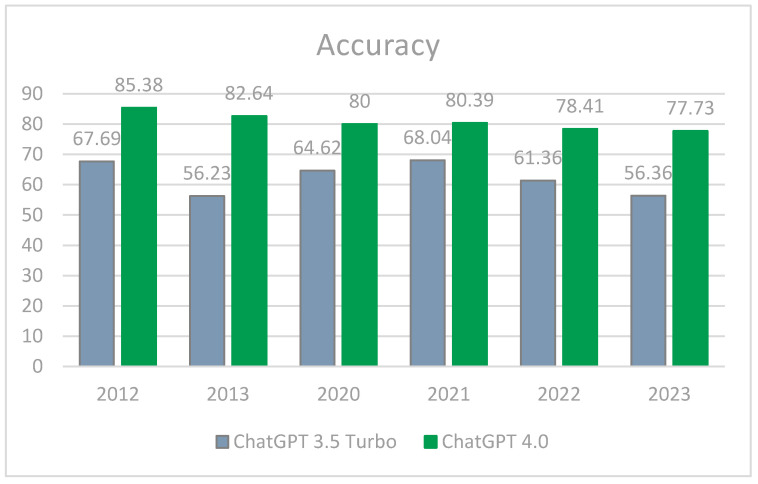
Comparison of accuracy scores for each exam paper.

After dividing all answers to MCQ questions in a 2 × 2 contingency table, we found a statistically significant difference in observed proportions vs. expected values for ChatGPT-3.5 Turbo and 4o (*p* < 0.001). ChatGPT-4o results were superior to 3.5 Turbo results across all metrics (80.40% vs. 63.18% accuracy, 84.62 vs. 60.97% recall, 80.77% vs. 68.76% precision, and 82.65% vs. 64.63% F1 score, respectively). ChatGPT-3.5 Turbo’s accuracy metrics did not change significantly, even after sitting several exams (R-squared values: 0.145 accuracy, 0.21 precision, 0.13 recall, and 0.16 F1), while ChatGPT-4o’s metrics decreased with each paper (R-squared values: 0.90 accuracy, 0.90 precision, 0.91 recall, and 0.96 F1) (Figure 1).

**Figure 2:**
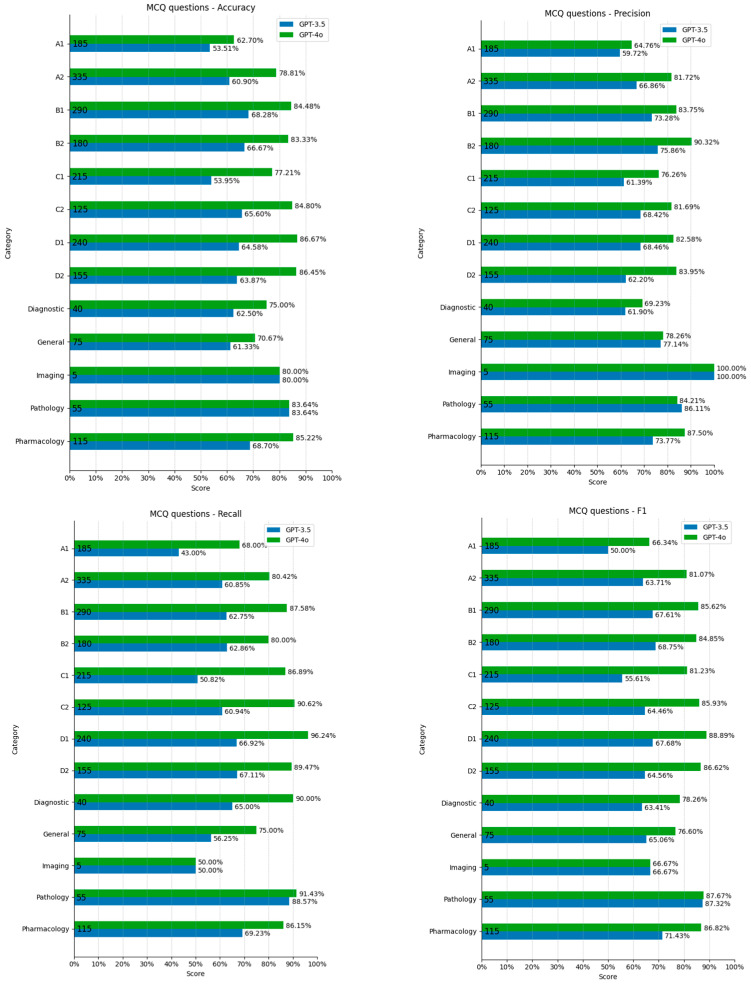
MCQ scores divided by category.

ChatGPT-3.5 Turbo’s accuracy was slightly higher for imaging and pathology and lower for glaucoma and optics/refraction questions, but there were with no significant outliers (Grubbs test *p* > 0.05), and R-square values were low (0.39 accuracy, 0.29 precision, 0.25 recall, and 0.38 F1). Pathology scores were significantly higher for precision and recall (Grubbs test *p* < 0.05) (Figure 2). Similarly, ChatGPT-4o’s metrics were fairly similar across the question categories (0.09 accuracy, 0.16 precision, <0.01 recall and 0.02 F1). The only significant outlier was lower recall in imaging questions (Grubbs test *p* < 0.05) (Figure 2).

### 3.2. Single Best Answers (SBAs)

**Figure 3:**
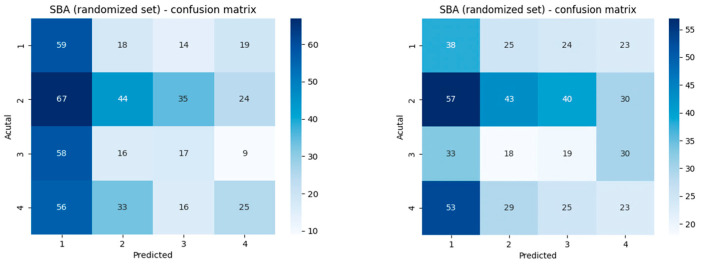
ChatGPT-3.5 Turbo (left) and ChatGPT-4.o (right) confusion matrixes for randomized SBA questions.

ChatGPT-3.5 Turbo selected the first answer in most cases, whether it was correct or not (*p* = 0.02119), even when having prior knowledge of the questions. ChatGPT-4o showed a similar tendency, though it was not statistically significant (*p* = 0.41) (Figure 3).

ChatGPT 3.5-Turbo’s metrics were higher than those of ChatGPT-4o when answering SBA questions (28.43% vs. 24.12% accuracy, 28.94% vs. 24.13% recall, 29.36% vs. 24.42% precision and 26.97% vs. 23.51% F1 respectively).

**Figure 4:**
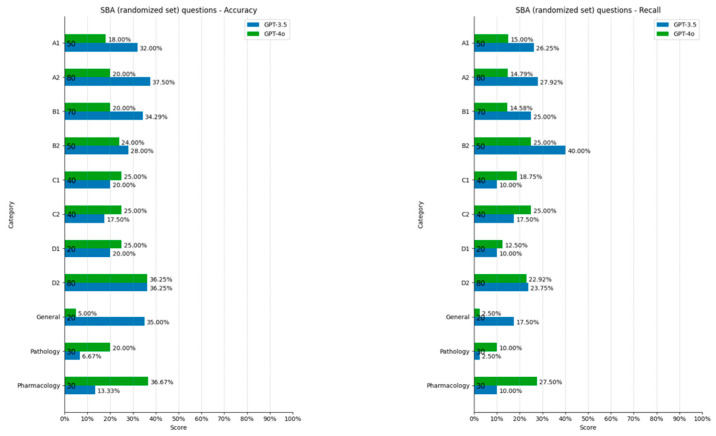

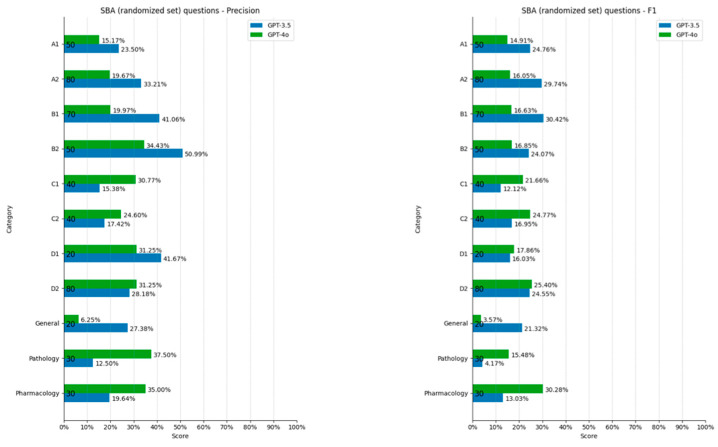
SBA scores divided by category.

ChatGPT 3.5-Turbo accuracy for pathology SBAs was lower (6.67%) than that for other categories, though the difference was not statistically significant (Grubbs test *p* > 0.05, R-squared 0.32). This was followed by pharmacology (13.33%) and cataract surgery (17.5%). Strabismus, pediatric ophthalmology, and neuro-ophthalmology were the categories of questions answered with the best accuracy (37.5% correct response rate.) This was followed by uveitis and general medicine, with 36.25% and 35% of questions answered correctly. For the other metrics, the highest scores were recorded for oculoplastics questions (precision, recall), and the lowest, for pathology questions (F1, recall), though these values were not statistically significant outliers (Grubbs test *p* > 0.05, R-squared precision = 0.16, R-squared recall = 0.45, R-squared F1 = 0.47) (Figure 4). 

ChatGPT-4o accuracy was lowest for general ophthalmology questions (5%) but it was not statistically significant. Uveitis and pharmacology questions were answered with the best accuracy (R-squared = 0.06, 36.25% and 36.67%, respectively) and high scores also in the other metrics. Scores were higher for oculoplastics (precision, recall), glaucoma (recall, F1), pathology (precision) but these values were not statistically significant outliers (Grubbs test *p* > 0.05, R-squared precision = 0.148, R-squared recall = 0.0002, R-squared F1 = 0.043).

### 3.3. Comparison with Candidates’ Performance

**Figure 5:**
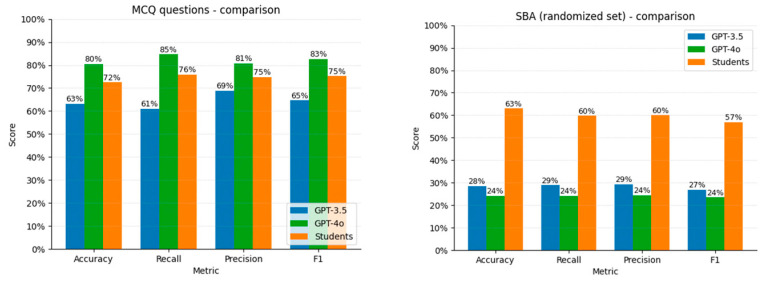
Candidate performance vs. ChatGPT metrics.

Our study used question sets that have been administered to candidates, so it was possible to directly compare the performance of ChatGPT-3.5 Turbo and 4o against that of 2935 candidates sitting the exam. For MCQ questions, candidates achieved better scores than ChatGPT-3.5 Turbo but not 4o for each quality metric. Conversely, candidates performed significantly better than ChatGPT-3.5 Turbo and 4o when answering SBAs (Figure 5).

**Figure 6:**
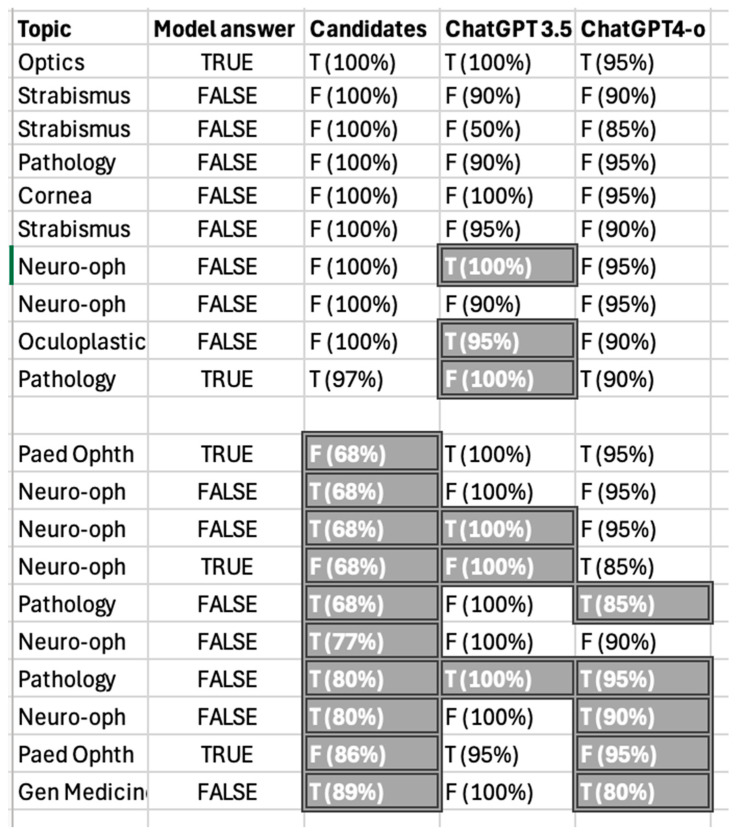
ChatGPT performance when answering the easiest and most difficult MCQs.

When looking at the top easiest questions, ChatGPT-4o answered them all correctly, while 3.5 Turbo managed only 70% of them. Conversely, ChatGPT-3.5 Turbo performed better than 4o in answering the most challenging questions (70% correct answers vs. 50%) (Figure 6).

## 4. Discussion

Our study shows that ChatGPT has improved its performance in a short period of time and that its latest version (ChatGPT 4-o) has the level of knowledge required to pass the EBO Diploma MCQ paper. On the other hand, it is significantly outperformed by human candidates when answering SBA questions.

### 4.1. MCQ Performance

ChatGPT-3.5’s Turbo performance answering MCQs was fairly consistent across each exam paper, and we did not demonstrate any learning effect, even after repeating the questions 10 times. It achieved scores above the minimum pass mark (60%) in five exam papers out of seven and scored very close to the pass mark otherwise. However, it was outperformed by the majority of human candidates and ChatGPT-4o. This was in line with other papers as well [10,19].

The main advantage of ChatGPT-4o over previous versions is its native multimodality. However, we excluded questions with tables and images from our study, so multimodality is unlikely to have played a role. We can also exclude a learning effect, because we did not share the correct answers with our account, leaving us with the most plausible explanation that that any performance gains were correlated to ChatGPT-4o’s superior computational efficiency [20].

Given that the difficulty and the format of each exam paper were similar, we wanted to test whether variations in performance were due to the way questions were formulated. It has been suggested that ChatGPT performs better on questions that humans found easier [21] but worse on ambiguous questions. In our study, ChatGPT-3.5 Turbo performed worse than human candidates on easy questions (75% vs. 100% accuracy). One example of a question that 100% of candidates answered correctly but ChatGPT did not was “*Spontaneous resolution of congenital naso-lacrimal blockage occurs rarely, so probing should be done as soon as possible* T/F”. On the other hand, ChatGPT-3.5 Turbo outperformed humans on challenging questions (50% vs. 28% accuracy), such as “*Opening CSF pressure > 20 cmH_2_O is included in the modified Dandy criteria for idiopathic intracranial hypertension in adults* T/F”, showing that ChatGPT could retrieve simple information accurately while most candidates confused 20 with 25 cmH_2_O. ChatGPT-4o answered easy questions without difficulty but it struggled with “In vivo *confocal microscopy can image non-filamentous fungi* T/F”, which was answered correctly by ChatGPT-3.5 Turbo. A possible explanation could be that ChatGPT-4o’s key features (ability to handle various modalities such as images and audio, more nuanced conversations, and superior accuracy in language generation tasks) did not provide an advantage in our study conditions, since all our MCQs were text-based and reliant on knowledge retrieval rather than language generation. Further studies are required to explore whether there is a trade-off between natural language processing and accuracy and consistency.

### 4.2. Performance Across MCQ Categories

When breaking down the scores for each category, our study shows that ChatGPT-3.5 Turbo performed better answering MCQs in pathology and imaging. The lowest scores were found for glaucoma and optics and refraction. A strong performance in pathology questions was repeated for the other metrics. This was an interesting finding, as all pathology and imaging questions in our exam papers were text-only. It is widely accepted that older versions of ChatGPT cannot process information presented as graphs and tables even though deep learning models have been successfully used to interpret clinical images in ophthalmology [22,23]. However, when presented with text-only pathology and imaging questions, ChatGPT-3.5 Turbo demonstrated excellent accuracy in determining if a statement was true or false.

ChatGPT-4o’s performance was strong across all categories, with pass rates achieved comfortably in 12 out of 13 categories and the highest scores in answering retina and uveitis questions (86.67% and 86.45, respectively), followed by questions on pharmacology (85.22%), the cornea (84.48%), and glaucoma (84.80%).

### 4.3. SBA Performance

The performances of both ChatGPT-3.5 Turbo and ChatGPT-4o were well below the pass mark for SBA questions, and we considered several explanations for this. First, our study showed that this ChatGPT version was biased towards selecting the first answer. To test this, we submitted the same exam paper multiple times but randomly changed the order of the possible answers, and the bias became even more obvious. ChatGPT-4o showed a similar tendency, but the bias was not statistically significant. Moreover, our findings align with previous studies suggesting that ChatGPT performs better at recalling facts or data rather than interpreting clinical scenarios [13,24]. It follows that questions that test higher-order knowledge (such as SBAs) challenged ChatGPT more than true/false MCQs. The EBO introduced SBA questions in 2022, so the chatbot was tested on a relatively smaller number of questions. SBA questions were also designed to test higher-order learning such as discriminating among differential diagnoses, each of them plausible. These questions cannot be answered using a process of elimination. Finally, a possible explanation for this less-than-satisfactory performance could be found in the choice of questions used. Our study was the first to use complete sets of original exam questions. In contrast, previous studies have extracted questions from question banks [8,9,11,13,14], and self-assessment programs [9,10,13] or used sample questions taken from the web [15]. We suggest that these studies’ results must be interpreted with caution, as they might not be a true representation of exam conditions. Panthier and Gatinel presented their results of ChatGPT performance on the French version of the EBO examination [11]. It should be, however, clarified that their work was based on a question bank created in 1998 by French University professors of ophthalmology but not endorsed by the EBO itself. In addition, since the questions were formulated in a different language, we cannot automatically extrapolate those findings to exams conducted in English. Most importantly, the authors did not divide their results by question type, jumbling together true/false questions, SBAs, and short-answer questions, making any comparisons challenging.

### 4.4. Performance Across SBA Categories

ChatGPT-3.5 Turbo’s performance in answering pathology SBA questions was poor, which was diametrically opposite to its performance with MCQ questions. ChatGPT-4o did better in pathology (20% vs. 6.67%) but performed worse in general medicine. While ChatGPT-3.5 Turbo answered questions with the greatest accuracy in the strabismus, pediatric ophthalmology, and neuro-ophthalmology categories (37.5%), ChatGPT-4o performed best in uveitis (36.25%) and pharmacology (36.67%) compared with the other categories. Previous papers have also reported low accuracy in answering neuro-ophthalmology and optics questions, with better accuracy in answering general medicine questions [8,9].

### 4.5. Comparison with Previous Studies

Antaki and colleagues opined that training data in general medicine could be more widely available. In their paper they report a better performance across all categories for ChatGPT Plus and we suggest that this supports the hypothesis that the chatbot version is closely linked to its performance in answering exam papers. The Authors also suggest that some categories are intrinsically more challenging, even for humans, quoting neuro-ophthalmology and pathology as examples [9]. It is therefore interesting to see that chatbots did relatively better than humans in this type of question in our study. A direct comparison of their findings with ours would not be possible, due to significant differences in sample size and study design (e.g., EBO exam questions are not available on the web while Antaki and colleagues used online question banks).

Our study is the first to be conducted on original and complete sets of EBO questions. We compare ChatGPT performance to humans in exam conditions, rather than during the preparation phase. We administered a larger number of questions compared to other studies. Because exam papers are designed to have an equal proportion of easy/intermediate/difficult questions, we can conclude from comparing papers from several years that differences in ChatGPT performance are due to the presence of biases and its limitations in higher-order learning. Finally, the questions are unavailable on the web, so we can rule out that ChatGPT has been trained on these questions. Excluding questions that contained images is a limitation of our study, even though this affected only a small amount of SBAs.

## 5. Conclusions

ChatGPT can understand and answer questions specific to the EBO diploma exams, showing an understanding of clinical ophthalmology comparable to that of a newly qualified specialist. ChatGPT achieved a pass mark for true/false questions in most instances, but its performance was poorer for SBA questions, showing that the chatbot can retrieve information better than it can integrate new knowledge. It is also possible for ChatGPT to be trained on biased or incorrect data, which could lead to errors. Interestingly, we found that ChatGPT-3.5 Turbo outperformed humans and ChatGPT-4o on challenging questions.

We believe that AI can be a valuable tool in ophthalmic education, for instance, allowing Exam Boards to test their exam papers with LLM to ensure that the questions are easily understood and are pitched at the right level of complexity. An appropriately trained LLM could also help with marking the papers, especially open-ended questions. Marking open-ended questions is challenging because of the variability in correct answers and subjectivity in marking, making it a time-consuming process [25]. A LLM that can grade these questions appropriately would also be able to provide detailed feedback, including explanations and suggestions on what do to next. Further research is needed to explore ChatGPT’s ability to generate exam questions or provide feedback to trainees. However, judging from the pace of the evolution of the chatbot, that moment could be just around the corner.

## Data Availability

The original contributions presented in this study are included in the article. Further inquiries can be directed to the corresponding author.

## Abbreviations

The following abbreviations are used in this manuscript:

EBO	European Board of Ophthalmology
MCQ	Multiple choice question
SBA	Single best answer
